# Sequential Limb Lengthening: A Novel Staged Approach Combining External Fixation and a Motorized Intramedullary Lengthening Nail for a Large Posttraumatic Tibial Defect

**DOI:** 10.1155/cro/2140595

**Published:** 2026-05-15

**Authors:** T. Cerasoli, G. Balboni, C. Musiani, M. Zaffagnini, A. Burla, G. M. Marcheggiani Muccioli, S. Zaffagnini, M. Romagnoli

**Affiliations:** ^1^ Department of Biomedical and Neuromotor Sciences DIBINEM, Alma Mater Studiorum University of Bologna, Bologna, Italy, unibo.it; ^2^ Ortopedia e Traumatologia Rizzoli Argenta, Rizzoli Orthopaedic Institute, Argenta (FE), Italy, ior.it; ^3^ 2nd Orthopaedic and Traumatologic Clinic, IRCCS Rizzoli Orthopaedic Institute, Bologna, Italy

**Keywords:** distraction osteogenesis, external fixation, limb lengthening, motorized intramedullary nail, sequential bone lengthening, staged limb reconstruction

## Abstract

**Background:**

Managing large posttraumatic tibial defects presents significant challenges. External fixation is effective for distraction osteogenesis but has drawbacks, including prolonged treatment time and complications. Intramedullary lengthening nails improve patient comfort but have limitations in large bone transport. While combined techniques have been explored, to our knowledge a fully sequential approach where an external fixator is first used for distraction and then replaced by a motorized intramedullary lengthening nail continuing the lengthening has not been clearly described in the literature. This study presents a novel staged technique and a review of existing literature on limb lengthening strategies.

**Methods:**

We report the case of a 29‐year‐old male with a 16‐cm posttraumatic tibial defect, treated with 10 cm of bone transport using an external fixator, followed by 6 cm of additional lengthening with a motorized intramedullary nail. Additionally, a literature search was conducted in PubMed, Scopus, and Embase using keywords related to limb lengthening, external fixation, and intramedullary nailing. The technique and outcomes were compared with existing literature.

**Discussion:**

The review did not identify any studies on fully sequential limb lengthening. Most studies focus on pediatric cases or hybrid techniques where external fixation and intramedullary nailing are used simultaneously. Our sequential approach allowed for controlled distraction and successfully addressed a 16‐cm bone defect.

**Conclusions:**

This case highlights a previously undescribed staged limb lengthening approach. Sequential conversion from external fixation to a motorized intramedullary lengthening nail may offer advantages in reducing EFI, protecting neurovascular structures, and enhancing mechanical stability. Further research is needed to evaluate long‐term outcomes.

## 1. Introduction

Managing posttraumatic limb length discrepancies is a complex challenge in orthopedic surgery, and it represents a long‐standing challenge long acknowledged in orthopedic practice [1]. Various techniques have been developed to achieve bone regeneration and restore limb function, including external fixation systems (such as the Ilizarov method) and intramedullary lengthening nails [2]. However, each approach has limitations: External fixators allow for effective bone transport and correction of large defects, but prolonged use is associated with patient discomfort and complications [3, 4]. Conversely, internal lengthening nails improve patient comfort but are generally limited in their ability to manage extensive segmental bone loss [5, 6].

To optimize treatment outcomes, combined techniques using both external fixators and intramedullary implants have been explored. However, the majority of the literature on this approach focuses on pediatric patients, where flexible intramedullary nails are used alongside external fixation to improve stability, enhance bone regeneration, and reduce the duration of external fixation [7–9].

Despite the growing interest in optimizing limb lengthening techniques, no studies in the literature have described a sequential approach where a fixator is initially used for distraction and later substituted with a lengthening nail to continue the process. This case report describes a novel staged limb lengthening approach for a 16‐cm posttraumatic tibial defect in an adult, using an external fixator for initial lengthening followed by a motorized intramedullary nail for continued distraction. This technique is aimed at reducing the duration of external fixation while optimizing bone regeneration, offering a potentially more effective strategy for managing large posttraumatic bone defects.

## 2. Case Presentation

**Figure 1:**
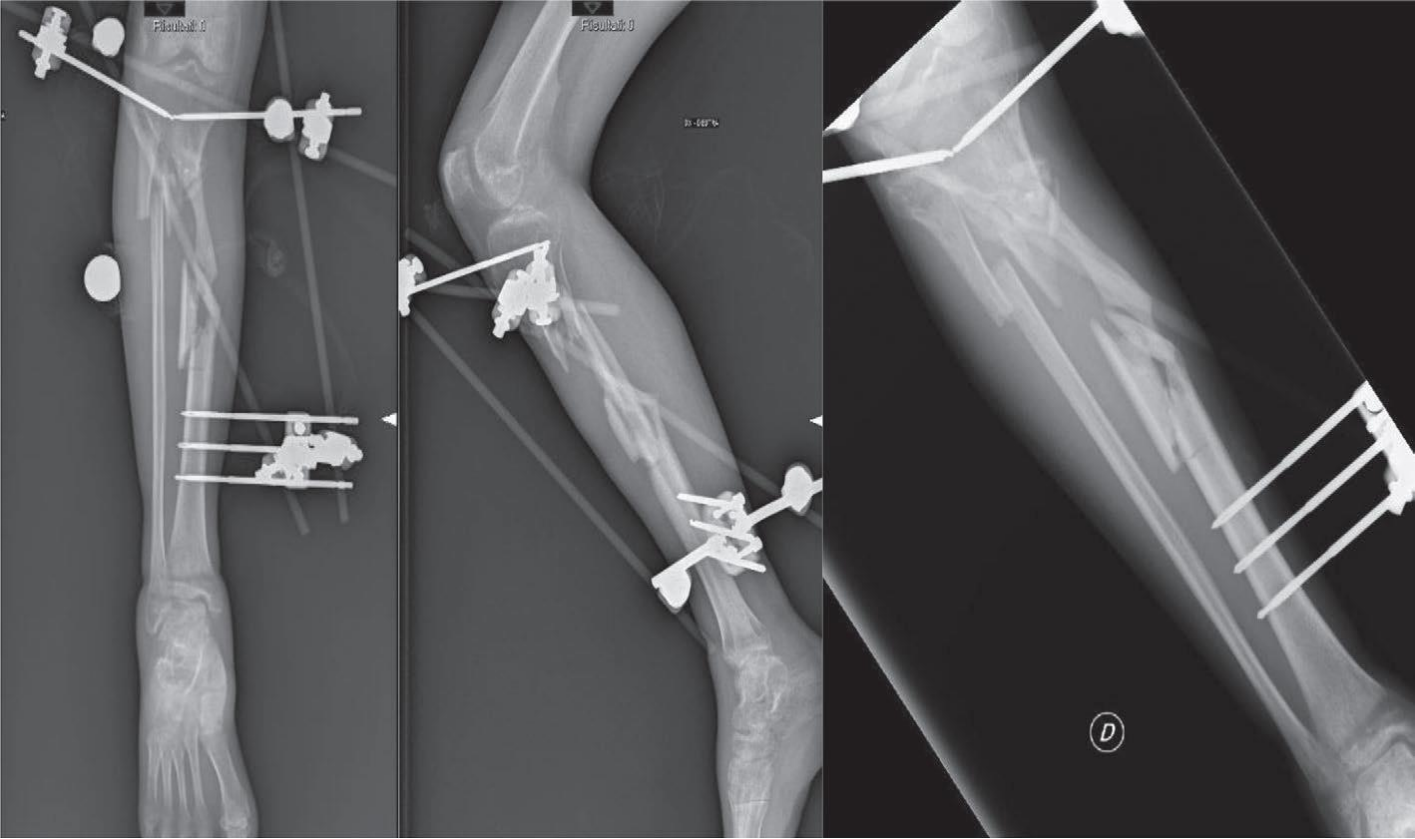
Initial patient presentation.

In April 2019, a 29‐year‐old male presented to our department after being treated for multiple fractures, including a complex open tibial fracture (Gustilo 3b), due to a motor vehicle accident in 2018. He presented with tibial nonunion still stabilized with the external fixator from the initial treatment (Figure 1).

**Figure 2:**
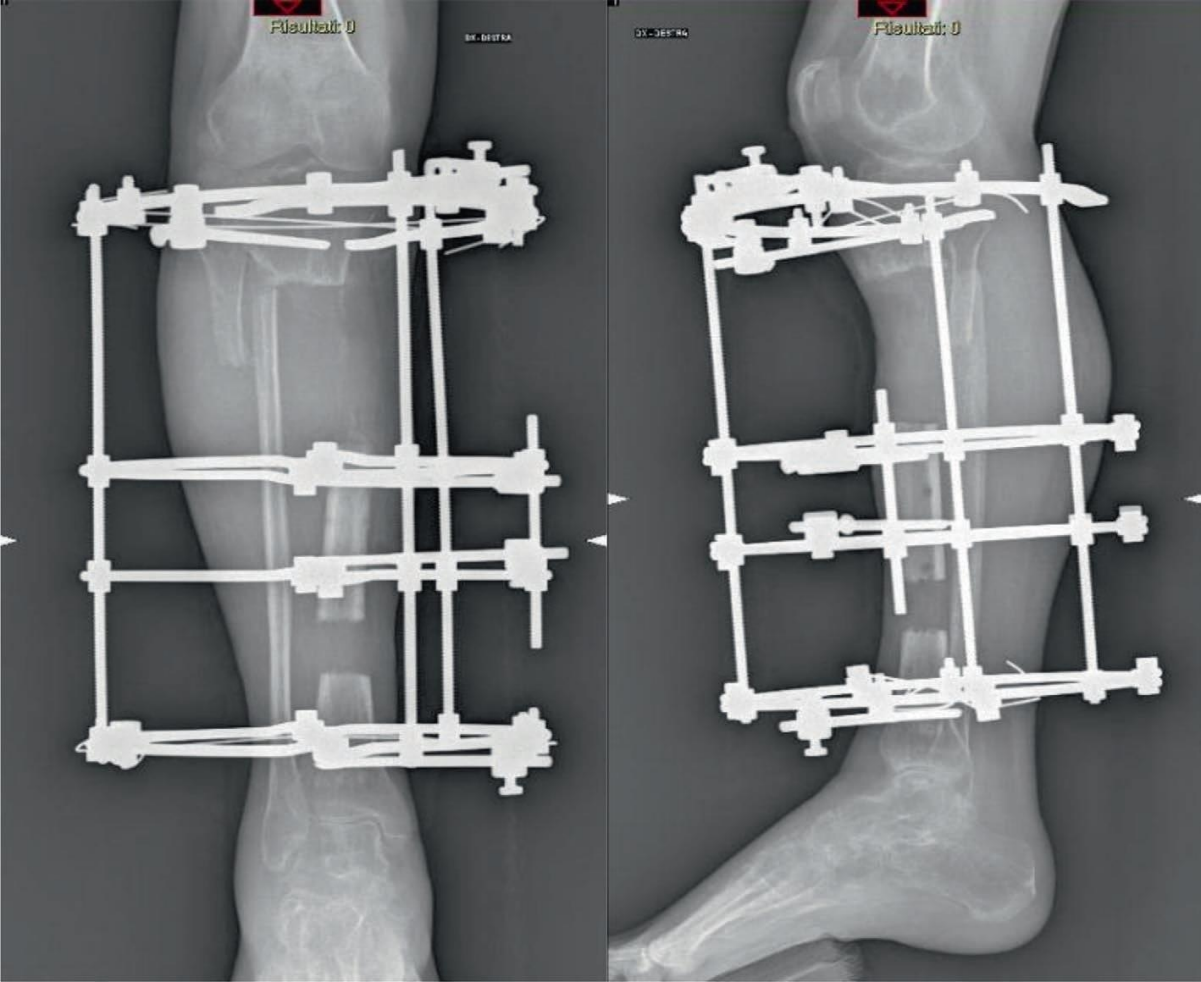
Ilizarov circular external fixator and distraction osteogenesis.

The patient underwent surgical debridement, removal of free bone fragments, and placement of an antibiotic‐laden cement spacer to partially fill the length defect and prevent infection. Despite no bacterial growth from cultures, a fistula developed, and the cement became mobilized in July 2019. A second debridement was performed, removing the fistula, the necrotic bone, and leaving a 16‐cm bone gap. The bacterial cultures at this time were positive for *Corynebacterium* species. The limb was stabilized with an Ilizarov circular external fixator, and distraction osteogenesis was initiated after antibiotic treatment (Figure 2).

By November 2019, early bone regeneration was observed. Further surgical intervention was performed to debride interposed soft tissues, clean the docking point, and compact the proximal tibial fragments, reducing the bone gap. Gradual bone transport was resumed in December 2019.

**Figure 3:**
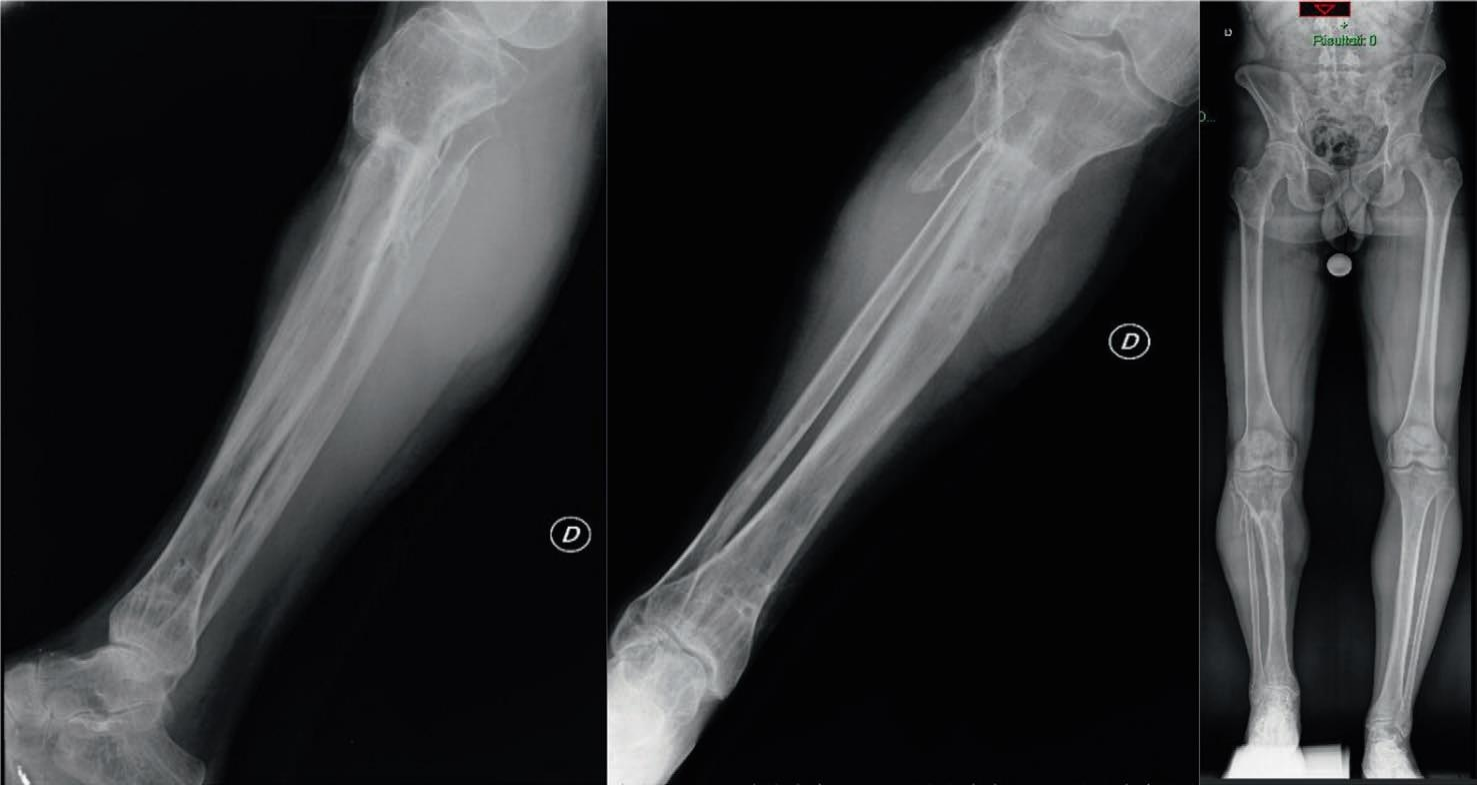
Patient′s condition after removal of FE, full‐length standing radiograph of the lower limbs with a 6‐cm lift on the right side.

In September 2020, after completing a 10‐cm bone transport phase, the external fixator was removed, and a cast brace was applied for 1 month. At that time, the patient still presented with a 6‐cm limb‐length discrepancy (Figure 3). At this stage, the ankle exhibited marked stiffness with a limited range of motion, particularly in dorsiflexion, while the knee maintained a ROM of 0°–120°. No neurovascular damage occurred throughout the procedure.

At that stage, the reconstruction was considered functionally acceptable, and no further surgical lengthening was initially planned. The patient had undergone a prolonged and psychologically demanding treatment period with the external fixator, and the ankle joint showed significant stiffness that might have required additional procedures in the future. For these reasons, a conservative approach with orthotic management and clinical follow‐up was adopted. During follow‐up, the regenerate remained stable without loss of length or signs of subsidence. However, due to persistent functional impairment related to the limb‐length discrepancy, the patient later requested further correction. After clinical reassessment and surgical planning, a second reconstructive stage was scheduled.

In the initial phase, external fixation had been necessary to manage infection and a large segmental bone defect, allowing safe bone transport and soft tissue management. However, repeating a further lengthening phase with an external fixator was considered poorly tolerated by the patient and potentially detrimental for the already compromised soft tissues. At the same time, the residual discrepancy was within the upper limits of what could be addressed with an intramedullary lengthening nail.

The interval between the two stages allowed complete consolidation and remodeling of the regenerate, which was therefore considered structurally mature at the time of the second procedure, enabling safe intramedullary reaming and stable nail insertion.

**Figure 4:**
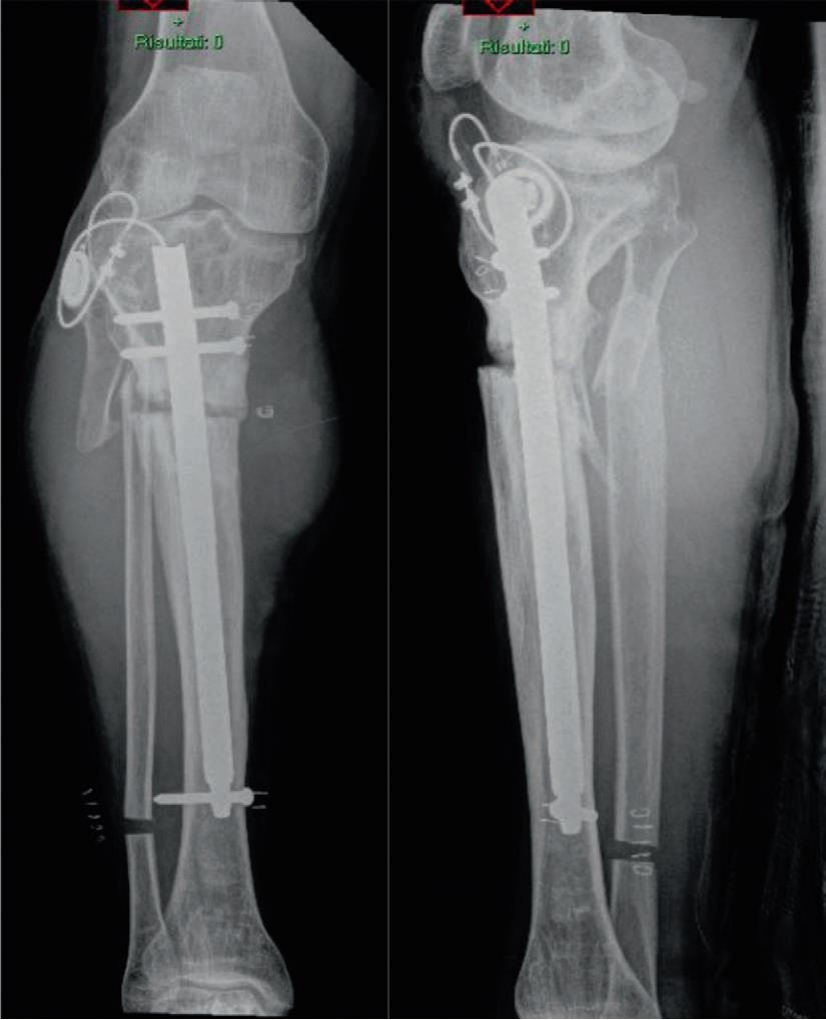
Radiograph showing the position of intramedullary lengthening nail with tibial and fibular lengthening osteotomy.

To address the residual 6‐cm limb‐length discrepancy, in November 2022, the patient underwent intramedullary nailing with a motorized lengthening nail (Orthofix Fitbone) and distal fibular osteotomy (Figure 4). The Fitbone system was selected to allow controlled and gradual distraction in the second stage, with reliable lengthening independent of soft tissue conditions. Progressive intramedullary reaming was performed according to the surgical technique, and the nail diameter was chosen to match the medullary canal and ensure adequate mechanical stability.

**Figure 5:**
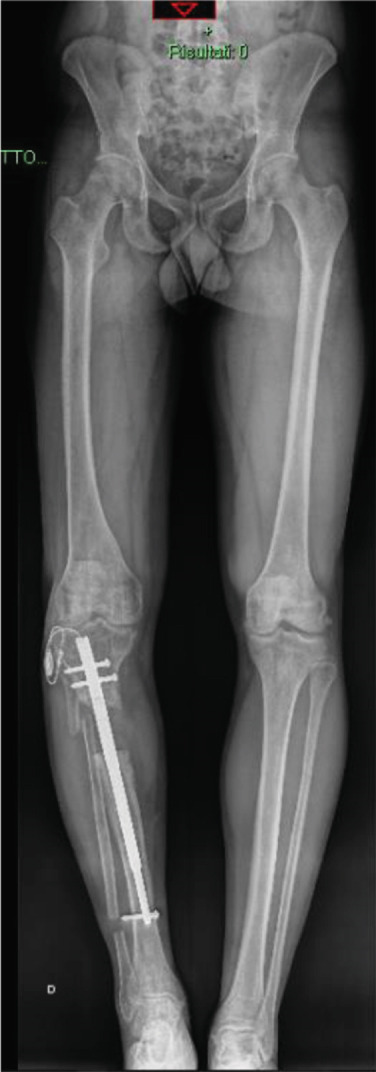
Radiograph showing progressive lengthening with motorized intramedullary lengthening nail.

Lengthening proceeded at a controlled rate, completing the distraction phase in 4 months. However, equinus contracture of the ankle persisted, requiring an Achilles tendon lengthening procedure and toe flexor tenotomy in May 2023. By September 2023, the patient demonstrated progressive weight‐bearing ability with near‐complete knee and ankle range of motion (Figure 5).

**Figure 6:**
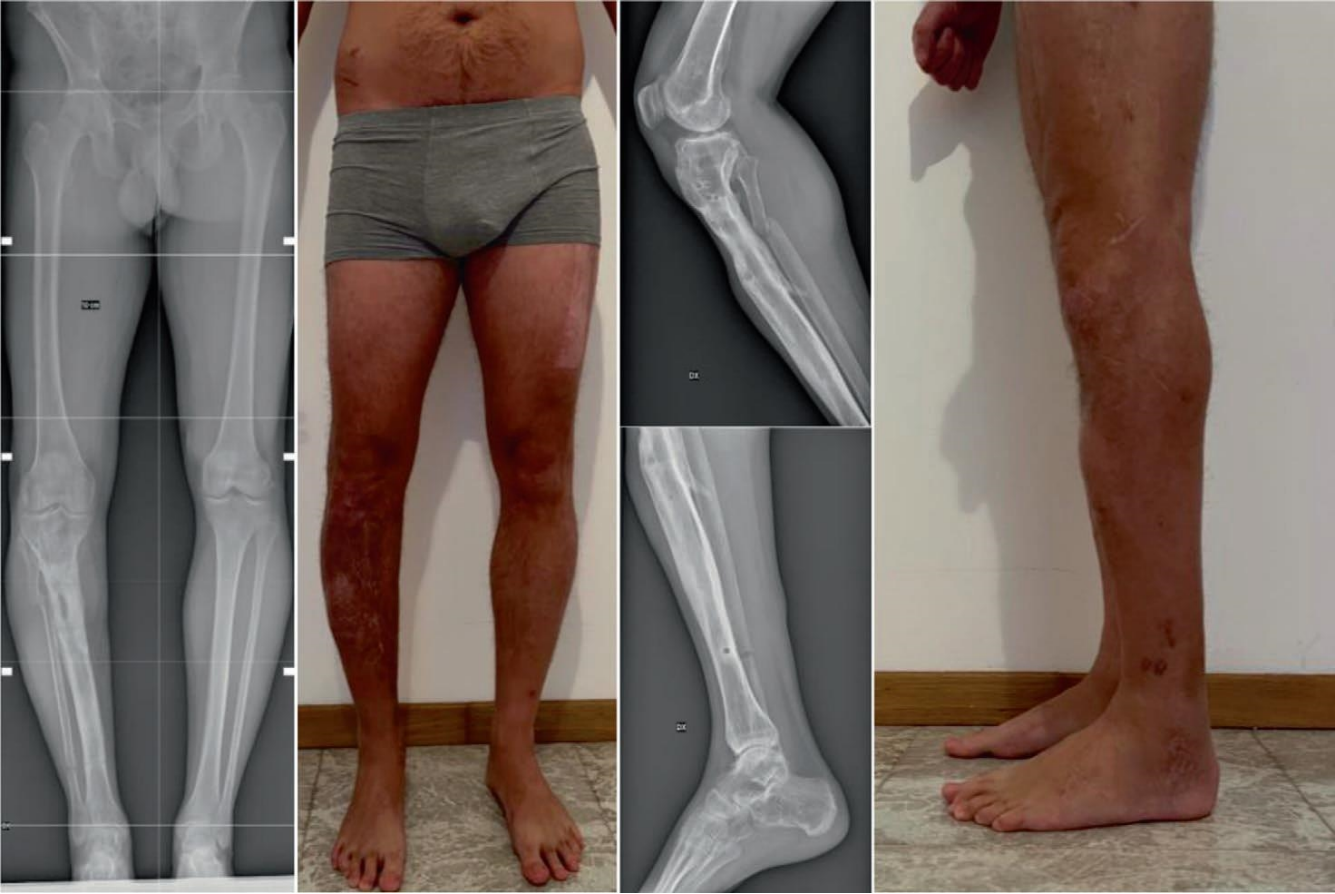
Composite panel showing: (left) full‐length anteroposterior standing radiograph of the lower limbs; (center) clinical photograph of the patient in standing position; (right, top) lateral knee radiograph; (right, bottom) lateral ankle radiograph; and (far right) lateral clinical photograph of the right lower limb at final follow‐up in September 2025.

Follow‐up imaging in 2024 showed ongoing consolidation of the regenerated bone. The patient underwent removal of the intramedullary nail in January 2025. By June 2025, he was regularly practicing stand‐up paddleboarding during the summer, swimming, table tennis, and cycling as recreational activities. Additionally, he attended the gym three times per week, performing mobility exercises, lunges, wall sits, and leg extensions. The most recent radiographs showed a residual limb length discrepancy of 1.3 cm on the right side, which the patient does not compensate for, as it is not perceived in daily life (Figure 6).

**Figure 7:**
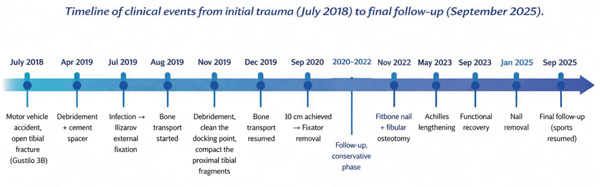
Timeline of clinical events from initial trauma (July 2018) to final follow‐up (September 2025).

The overall clinical course is summarized in the timeline shown in Figure 7.

## 3. Patient Perspective

The patient described the period with the external fixator as physically and psychologically demanding, mainly due to discomfort, limitations in daily activities, and the burden of device management. Despite these challenges, he remained compliant with treatment, motivated by the progressive improvement in limb function and supported by several favorable personal factors. The patient was young, otherwise healthy, and had a strong family support system, which played a crucial role, particularly during the early stages of treatment. Family members assisted him with hospital visits and follow‐up appointments and supported his rehabilitation process, including regular attendance at the gym.

After removal of the external fixator, the patient had reached a good level of functional independence and initially preferred to avoid further surgery. However, the residual limb‐length discrepancy progressively affected both function and quality of life, making activities such as walking, running, and rapid movements difficult. The need for a 6‐cm shoe lift was also poorly tolerated from a psychological perspective, as the patient felt uncomfortable and limited in social situations. These factors led him to request further correction. The second stage with the intramedullary lengthening nail required a period of partial weight‐bearing, temporarily reducing his autonomy; therefore, the presence of a supportive family environment played an important role in both the decision‐making process and postoperative management.

The intramedullary lengthening phase was perceived as less invasive and more manageable compared with external fixation, with improved comfort during daily activities. At final follow‐up, the patient reported high satisfaction with the overall outcome and was able to return to multiple recreational sports without significant limitations. However, he remains actively engaged in regular training and rehabilitation, aware that maintaining these results requires ongoing effort.

This case report was prepared in accordance with the CARE guidelines. A completed CARE checklist is provided as Supporting Information (available here).

## 4. Discussion

A narrative literature review was conducted to evaluate existing evidence on combined and staged limb lengthening techniques, using the following search strategy in PubMed, Scopus, and Embase: (“Limb Lengthening” OR “Bone Lengthening” OR “Distraction Osteogenesis”) AND (“External Fixation” OR “Ilizarov Technique” OR “Circular External Fixator”) AND (“Intramedullary Nailing” OR “Lengthening Nail” OR “Magnetic Intramedullary Nail”) AND (“Hybrid Technique” OR “Combined Lengthening” OR “Two‐stage Lengthening” OR “Staged Limb Lengthening” OR “Sequential Limb Lengthening”).

To our knowledge, no studies have clearly described a fully sequential approach in which an external fixator is used first for distraction and subsequently replaced by a motorized lengthening nail for continued distraction. Instead, the existing literature primarily discusses combined approaches, where external fixation and intramedullary nailing are used simultaneously rather than in distinct phases.

These findings highlight the current gap in the literature regarding staged limb lengthening techniques. Although hybrid approaches have been widely reported, they are predominantly used in pediatric patients with congenital or metabolic bone disorders, where flexible intramedullary nails are combined with external fixation to reduce fixation time and enhance bone regeneration [7, 9, 10]. However, these cases do not involve staged lengthening, as both systems are applied at the same time rather than in a sequential manner.

In adult patients, hybrid strategies combining external fixation and intramedullary devices have previously been described, although with different biomechanical roles for each implant [11–15]. Fragomen et al. reported the transition from external fixation to a motorized intramedullary lengthening nail, where the external fixator was primarily used for temporary stabilization in the acute trauma setting, whereas the intramedullary nail was subsequently used as the active lengthening device [15]. In contrast, the “lengthening and then nailing” (LATN) technique described by Rozbruch et al. follows the opposite principle: Distraction osteogenesis is performed with external fixation, and the intramedullary nail is inserted afterward to stabilize the regenerate and allow earlier removal of the frame [14]. The strategy described in the present case differs from both approaches. In our patient, the circular external fixator was initially used to perform bone transport for reconstruction of the segmental defect, whereas the motorized intramedullary nail was subsequently used to achieve additional lengthening after consolidation of the transported segment. Moreover, most published cases involve relatively small bone defects, with reported lengthening typically limited to 5 cm or less [2, 4, 6]. In contrast, our case required 16 cm of total lengthening, demonstrating the feasibility of a sequential approach for managing large posttraumatic tibial defects. A summary of the main limb lengthening strategies and their key characteristics is provided in Table 1.

**Table 1:** Overview of limb lengthening strategies and their key characteristics, including underlying principles, advantages, and limitations.

Technique	Principle	Advantages	Limitations
External fixation alone	Distraction osteogenesis using circular or monolateral fixators	Suitable for large defects, allows infection control and soft tissue management	Long treatment duration, high external fixation index (EFI), discomfort, pin‐site complications
LATN (lengthening and then nailing)	External fixation for distraction followed by intramedullary nail for stabilization	Reduces time in external fixation, improves stability during consolidation	Nail does not contribute to lengthening, requires two procedures
Hybrid techniques (simultaneous)	External fixation combined with intramedullary device during lengthening	Increased stability, potential reduction of complications	Technically demanding, mainly described in pediatric populations
Sequential approach (present case)	Initial bone transport with external fixation followed by intramedullary lengthening	Reduces external fixation duration, improves patient comfort, allows treatment of large defects	Limited evidence, technically demanding, requires careful patient selection

This sequential lengthening approach allowed for an initial distraction of 10 cm using an external fixator, followed by an additional 6 cm of lengthening with a motorized intramedullary nail. One of the key advantages of this technique is the ability to control the rate of lengthening, ensuring a slow and gradual distraction process, which is particularly important for nerve protection. Rapid distraction increases the risk of neuropraxia or permanent nerve damage, especially in cases involving extensive defects. By progressing in a controlled manner, our approach reduced the likelihood of neurological complications, providing an additional safety advantage over traditional methods. Unlike magnetically driven lengthening nails, the Fitbone system is based on an electromechanical motor powered through transcutaneous induction. This mechanism provides reliable distraction forces that are independent of soft tissue thickness and may be advantageous in complex reconstructions requiring substantial length restoration.

Another major benefit of this method is its impact on the external fixation index (EFI), a critical parameter in patient recovery. The EFI is directly related to the duration of external fixation per centimeter of length gained; reducing this index has been shown to lower the risk of pin‐site infections, muscle contractures, and joint stiffness [5]. By transitioning to an internal lengthening nail after 10 cm of distraction, we effectively shortened the duration of external fixation, improving patient comfort and potentially reducing long‐term morbidity.

Moreover, intramedullary stabilization in the second phase of treatment provided additional mechanical support, reducing the risk of bending or refracture during the consolidation phase. While some studies have investigated the use of internal fixation after external fixation–assisted lengthening [16], these approaches have generally been applied in a nonsequential manner, without utilizing an intramedullary nail as an active lengthening device in the second phase.

A crucial advantage of this sequential approach is its role in infection management, a key concern in cases of open fractures with large bone defects. External fixation serves as the primary treatment modality in the acute phase, allowing for better infection control by avoiding the implantation of an intramedullary nail in a contaminated surgical field. External fixation remains the gold standard for managing open fractures with bone loss, providing stability while facilitating wound care, soft tissue management, and staged debridement. In contrast, immediate intramedullary nailing in an infected or potentially contaminated environment carries a high risk of deep infections, which can severely compromise bone healing and patient outcomes [17]. By delaying the use of the intramedullary nail until after infection risks have been mitigated, our approach enhances overall treatment safety while optimizing bone regeneration [6].

Despite the growing interest in optimizing limb lengthening techniques, no studies in the literature describe a fully sequential approach, where an external fixator is first used for distraction, followed by conversion to a motorized lengthening nail for continued distraction. This absence of published data highlights the novelty of our technique, which may offer a promising alternative for reducing EFI, protecting neural structures, and maintaining mechanical stability throughout the lengthening process.

Further research is needed to evaluate long‐term outcomes, bone healing rates, and functional recovery in a larger patient cohort. Comparative studies should also assess whether this staged strategy provides superior bone regeneration and lower complication rates compared with traditional external fixation–only methods or fully internal lengthening techniques.

## 5. Conclusions

This case demonstrates the feasibility of a sequential limb lengthening approach, combining an initial phase of distraction osteogenesis with an external fixator followed by further controlled lengthening with an intramedullary nail. This staged technique represents a potential advancement in the treatment of large posttraumatic tibial defects by reducing the duration of external fixation, ensuring effective bone regeneration, and protecting neurovascular structures through a controlled lengthening process.

**Nomenclature**
EFI: external fixation index
EF: external fixation

## Author Contributions

Conceptualization: T.C. and C.M.; methodology: T.C.; software: A.B.; validation: M.R., S.Z., and G.M.M.M.; formal analysis: M.Z.; investigation: G.B.; resources: G.B.; data curation: T.C.; writing—original draft preparation: T.C.; writing—review and editing: M.R. and C.M.; visualization: G.B., A.B., and M.Z.; supervision: M.R. and S.Z.; project administration: M.R.; funding acquisition: M.R.

## Funding

No funding was received for this manuscript. Open access publishing facilitated by Universita di Bologna, as part of the Wiley ‐ CRUI‐CARE agreement.

## Disclosure

All authors have read and agreed to the published version of the manuscript.

## Ethics Statement

Ethical review and approval were waived for this study as it is a case report describing a single patient′s clinical course and treatment. According to institutional and international guidelines, case reports do not require formal ethical approval when no experimental interventions are performed, and the patient′s confidentiality is strictly maintained.

## Consent

Written informed consent has been obtained from the patient to publish this paper.

## Conflicts of Interest

The authors declare no conflicts of interest.

## Supporting information


**Supporting Information** Additional supporting information can be found online in the Supporting Information section. Supporting Information includes the CARE checklist used for the preparation of this case report.

## Data Availability

The data that support the findings of this study are available on request from the corresponding author. The data are not publicly available due to privacy or ethical restrictions.
