# Supplementary material for: Sequential Limb Lengthening: A Novel Staged Approach Combining External Fixation and a Motorized Intramedullary Lengthening Nail for a Large Posttraumatic Tibial Defect
Source: Case Rep Orthop. 2026 May 15;2026:2140595. doi: 10.1155/cro/2140595 (PMC13178156; doi:10.1155/cro/2140595)
Supplement: Supplementary file 1 — Supporting Information Additional supporting information can be found online in the Supporting Information section. Supporting Information includes the CARE checklist used for the preparation of this case report. [file CRO-2026-2140595-s001.pdf]

CARE case report guidelines

CARE Checklist of information to include when writing a case report

(cc) BY-NC-ND

Topic	Item	Checklist item description	Reported on Line
Title	1	The diagnosis or intervention of primary focus followed by the words "case report"	1
Key Words	2	2 to 5 key words that identify diagnoses or interventions in this case report, including "case report"	33-34
Abstract (no references)	3a	Introduction: What is unique about this case and what does it add to the scientific literature?	36-54
	3b	Main symptoms and/or important clinical findings	56-58
	3c	The main diagnoses, therapeutic interventions, and outcomes	77-186
	3d	Conclusion—What is the main "take-away" lesson(s) from this case?	279-283
Introduction	4	One or two paragraphs summarizing why this case is unique (**may include references**)	210-277
Patient Information	5a	De-identified patient specific information.	56-58
	5b	Primary concerns and symptoms of the patient.	58
	5c	Medical, family, and psycho-social history including relevant genetic information	56
	5d	Relevant past interventions with outcomes	56
Clinical Findings	6	Describe significant physical examination (PE) and important clinical findings.	77-82
Timeline	7	Historical and current information from this episode of care organized as a timeline	77-186
Diagnostic Assessment	8a	Diagnostic testing (such as PE, laboratory testing, imaging, surveys).	77-186
	8b	Diagnostic challenges (such as access to testing, financial, or cultural)	77-186
	8c	Diagnosis (including other diagnoses considered)	77-186
	8d	Prognosis (such as staging in oncology) where applicable	77-186
Therapeutic Intervention	9a	Types of therapeutic intervention (such as pharmacologic, surgical, preventive, self-care)	77-186
	9b	Administration of therapeutic intervention (such as dosage, strength, duration)	77-186
	9c	Changes in therapeutic intervention (with rationale)	82-186
Follow-up and Outcomes	10a	Clinician and patient-assessed outcomes (if available)	181-186
	10b	Important follow-up diagnostic and other test results	181-186
	10c	Intervention adherence and tolerability (How was this assessed?)	77-186
	10d	Adverse and unanticipated events	77-82
Discussion	11a	A scientific discussion of the strengths AND limitations associated with this case report	210-277
	11b	Discussion of the relevant medical literature **with references**.	210-277
	11c	The scientific rationale for any conclusions (including assessment of possible causes)	210-277
	11d	The primary "take-away" lessons of this case report (without references) in a one paragraph conclusion	279-283
Patient Perspective	12	The patient should share their perspective in one to two paragraphs on the treatment(s) they received	181-186
Informed Consent	13	Did the patient give informed consent? Please provide if requested	Yes ☐ No ☐
